# A Multi-Parameter Fusion Method for Cuffless Continuous Blood Pressure Estimation Based on Electrocardiogram and Photoplethysmogram

**DOI:** 10.3390/mi14040804

**Published:** 2023-03-31

**Authors:** Gang Ma, Jie Zhang, Jing Liu, Lirong Wang, Yong Yu

**Affiliations:** 1 School of Biomedical Engineering, Division of Life Sciences and Medicine, University of Science and Technology of China, Hefei 230026, China; 2 Suzhou Institute of Biomedical Engineering and Technology, China Academy of Sciences, Suzhou 215163, China; 3 School of Electronics and Information Technology, Soochow University, Suzhou 215031, China

**Keywords:** blood pressure, wearable device, feature extraction, GCMI

## Abstract

Blood pressure (BP) is an essential physiological indicator to identify and determine health status. Compared with the isolated BP measurement conducted by traditional cuff approaches, cuffless BP monitoring can reflect the dynamic changes in BP values and is more helpful to evaluate the effectiveness of BP control. In this paper, we designed a wearable device for continuous physiological signal acquisition. Based on the collected electrocardiogram (ECG) and photoplethysmogram (PPG), we proposed a multi-parameter fusion method for noninvasive BP estimation. An amount of 25 features were extracted from processed waveforms and Gaussian copula mutual information (MI) was introduced to reduce feature redundancy. After feature selection, random forest (RF) was trained to realize systolic BP (SBP) and diastolic BP (DBP) estimation. Moreover, we used the records in public MIMIC-III as the training set and private data as the testing set to avoid data leakage. The mean absolute error (MAE) and standard deviation (STD) for SBP and DBP were reduced from 9.12 ± 9.83 mmHg and 8.31 ± 9.23 mmHg to 7.93 ± 9.12 mmHg and 7.63 ± 8.61 mmHg by feature selection. After calibration, the MAE was further reduced to 5.21 mmHg and 4.15 mmHg. The result showed that MI has great potential in feature selection during BP prediction and the proposed multi-parameter fusion method can be used for long-term BP monitoring.

## 1. Introduction

Blood pressure (BP) is one of the most important parameters for monitoring the status of the body and can be used in the diagnosis and treatment of many diseases. Unfortunately, in recent years, the number of hypertension (HPT) patients continued to increase and became an urgent global health problem [1]. HPT could increase the pressure of blood on the walls of the arteries, which gradually affects several organs, including the heart, brain, and kidneys. HPT even has the potential to cause the blockage or rupture of blood vessels that supply oxygen to the brain, which greatly increases the risk of accidents [2,3,4]. BP regulation is influenced by a variety of factors, such as cardiac ejection, peripheral resistance, and vessel wall elasticity, and also has a relation with mood and age. As HPT is a chronic disease that requires long-term monitoring, a single BP measurement is not able to provide an accurate view of the body’s condition. Therefore, it is very important to propose a noninvasive continuous blood pressure estimation method, which can reflect the physical condition of the human body in real time and reduce the incidence of cardiovascular diseases [5].

The mainstream cuffless BP measurement methods analyze the features of physiological signals such as photoplethysmogram (PPG) [6], electrocardiogram (ECG) [7], ballistocardiogram (BCG) [8], etc., and predict BP by machine learning (ML). The key technologies are physiological signals acquisition [9] and feature extraction [10,11]. In the last decade, with the development of semiconductor technology and the popularity of wearable health monitoring devices, people were found to use such devices to achieve a variety of physiological signal acquisition without interfering with normal human activities. The quality of signals directly affects the accuracy of prediction. This poses a challenge to the design of acquisition hardware but also makes continuous BP monitoring possible. In [12], Rachim et al. designed a multimodal biosensor to measure PPG and impedance plethysmography (IPG) from the participant’s wrist. Then, 14 PTT-features were calculated between the IPG peak-point and PPG. The comparative experiment showed that the extracted PTT-features had a certain correlation with BP, but its correlation coefficient for SBP was weaker than that of conventional PAT, which was located by ECG. In addition, compared with the fingertip, the wrist was more active and had a bad influence on the collected PPG signals. Therefore, the author only analyzed the time characteristics and did not use other amplitude characteristics. Bui et al. [13] chose the ear as the measuring location to minimize motion interference and ensure comfort. Benefiting from the good contact between the ear and the sensor, the author collected the PPG signal with high quality. However, because of the lack of ECG as a reference, the features provided by a single PPG are still limited. Multiple types of signals are conducive to obtaining richer features and improving the accuracy of results. Hence, researchers must consider comfort, signal diversity, and quality while using wearable devices to collect physiological signals.

For feature extraction, scholars try to synthesize multiple physiological signals and select information in multiple dimensions such as the time domain and frequency domain. Among them, pulse transit time (PTT) [14], pulse arrival time (PAT) [15], pulse wave velocity (PWV) [16], etc. were proven to be effective. Chen W et al. [17] adopted that PTT was highly correlated with the high-frequency components of BP. Through calibration, more accurate blood pressure estimation can be achieved. Meanwhile, age, gender, cardiovascular disease, and other factors determine arterial stiffness, which is significantly correlated with PTT [18]. Zhang et al. [19] considered how the autonomic nervous system affects the heart and blood vessels. He extracted nine features related to heart rate variability (HRV) and combined them with other PPG features. The result proved the effectiveness of HRV features. Geerthy et al. [20] extracted informative features such as SDI, Womersley, and QRS from PPG and ECG signals and used a genetic algorithm (GA) to select features. The best optimal feature set reduced the mean absolute error (MAE) from 13.20 mmHg to 9.54 mmHg for systolic BP (SBP) and 9.91 mmHg to 5.48 mmHg for diastolic BP (DBP), respectively. Wang et al. [21] collected 30 sets of PPG and ECG signals by Finometer. Then, 39 features were extracted and 10 features were finally retained by comparing their correlations with BP. In [22], 32 features from PPG were extracted to estimate BP. These features almost comprehensively contained multiple scale information such as amplitude, time, and frequency. Shuo et al. [23] introduced the mean impact value (MIV) to investigate the impact of each feature and the genetic algorithm (GA) to implement parameter optimization. After optimization, the MAE of SBP and DBP reached 3.27 mmHg and 1.16 mmHg. Through the joint analysis of the PPG signal and its derivatives, Chowdhury et al. [24] extracted up to 101 features, among which 75 features were from the time domain, 16 were from the frequency domain, and the remaining 20 were computed using statistical analysis. It was observed that the number of features extracted from the signal increased, which not only aggravated the workload of feature extraction but also made model training more difficult. Compared with the feature extraction itself, how to screen out the optimal solution from many features became another issue for scholars to research.

Different from traditional ML methods, deep learning (DL) methods avoid the drawbacks of manual feature extraction. It can automatically learn more abstract and high-dimensional features from the input signal, which enables it to have stronger adaptability for nonlinear system complexity [11]. Benefiting from its powerful data mining capabilities, machine learning can be combined with biological signal processing and achieved good performance [25]. Yu et al. [26] introduced an attention-based residual block to U-Net to predict BP. The result showed that the combination of PPG raw signal, first derivative, and second derivative as model input was helpful for the network to extract more information. Wang et al. [27] proposed an end-to-end model to measure BP. The model consisted of one-dimensional convolutional layers, depth-separable convolutional layers, and a gated recurrent unit (GRU). The average absolute error (MAE) was 3.95 mmHg for SBP and 2.14 mmHg for DBP, which met the international standard. Senturk et al. [28] analyzed the performance of dynamic learning methods, such as recurrent neural networks (RNN), nonlinear autoregressive networks with exogenous inputs neural networks (NARX-NN), and long short-term memory neural networks (LSTM-NN). The experiment showed that NARX had the most potential.

Despite the great performance in BP prediction, DL still has the following shortcomings. On the one hand, it relies on a large amount of data during the training process, and it is difficult to achieve good performance on small batches of data. On the other hand, neural network features are not easily understood at the semantic level, which makes it more challenging for scholars to interpret the experimental results.

Above all, as ECG and PPG signals have irreplaceable effects on BP assessment, we designed a wearable physiological signal acquisition device that could continuously collect and transmit two signals in real time. In order to make the acquisition process comfortable, we adopted a separate design for the host and the sensor. Meanwhile, combining individual features with waveform features, a multi-parameter fusion method for non-invasive continuous BP estimation was proposed and mutual information (MI) [29] was introduced for feature selection. The main contributions in this paper are as follows:

(1) We used a split design where the acquisition host and sensor were connected via a type-C interface. This not only ensured the quality of the collected signal but also facilitated the replacement of the sensor. The host can either transmit data wirelessly in real time or store data locally as a backup. The whole host size was only 42 mm × 29 mm × 13 mm and caused a little burden on the human body.
(2) We constructed a private database by self-collected data. Then, the model was trained on the MIMIC III dataset [30] (accessed on 10 January 2020) and tested on the private dataset, which avoided data leakage. After training, we calibrated the model with a quarter of the records in the testing set.
(3) Gaussian copula MI (GCMI) was used to rank the initial 25 features. Then, 11 and 15 features were retained for SBP and DBP prediction, respectively. The results showed that the optimal feature set improved performance.

## 2. Data Preparation

In this section, we will describe the wearable device structure and the data acquisition process for noninvasive continuous BP estimation.

### 2.1. Cuff-Less Continuous BP Measurement System

**Figure 1:**
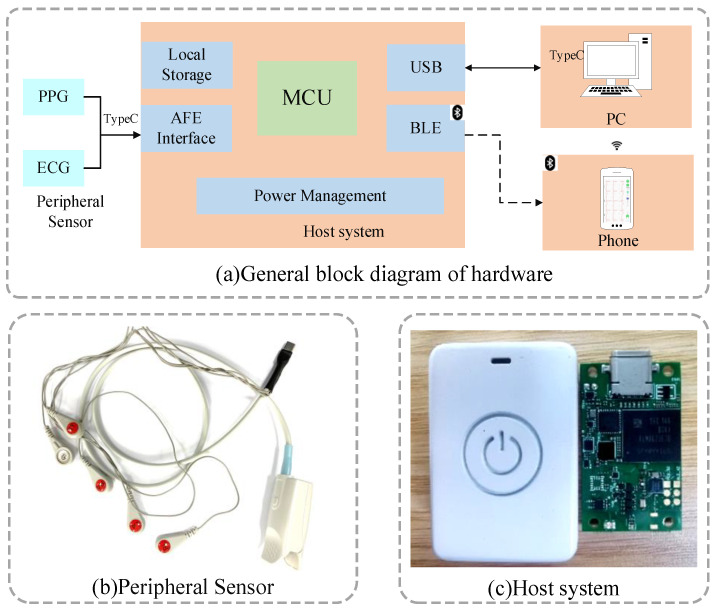
Hardware system. (**a**) General block diagram of hardware; (**b**) Peripheral sensor; (**c**) Host system.

The hardware block diagram of the BP monitoring system designed in this paper is shown in Figure 1a. The host system consisted of five parts: the main control unit, the signal acquisition front-end (AFE) circuit, the storage circuit, the wireless transmission circuit, and the power supply management circuit. The STM32F205 chip was chosen as the main control, which had an Arm Cortex-M3 core with a maximum main frequency of 120 MHz. The acquisition circuit contained the synchronous record of PPG and ECG signals. For ECG signals, ADS1298 was selected as the AFE chip to collect the lead II signals. It could provide 24-bit analog-to-digital conversion accuracy and up to 12× signal amplification. At the same time, it had a 500 Mohm input impedance and ±400 mV dynamic input range, which met the requirements of ECG acquisition. For PPG signals, AFE4490 was used as the analog AFE. It had two programmable 8-bit resolution current output functions. The maximum output current was 200 mA and could drive red light and infrared light. For the convenience of acquisition, we designed a peripheral sensor that integrated a finger-clip blood oxygen probe and ECG electrodes, which were connected to the main circuit through a type-C interface. The sampling rate was set to 125 Hz. We chose nRF52832 as the wireless transmission chip, which was equipped with Bluetooth Low-power Energy (BLE) wireless communication protocol and can communicate with other Bluetooth devices. Meanwhile, considering the instability of wireless transmission, we selected a 32 GB eMMC as the local storage, which was suitable for long-term collection.

The whole hardware system is shown above. Figure 1b exhibits the customized ECG electrodes and oximetry probe and Figure 1c exhibits the final host device and the internal PCB circuit.

### 2.2. Data Collection

**Figure 2:**
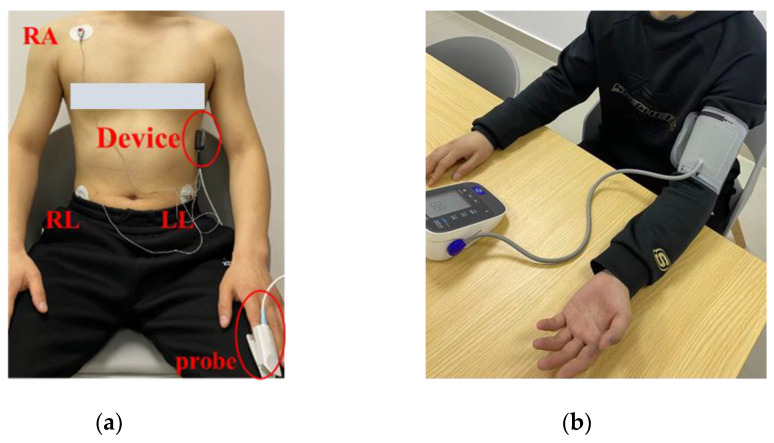
Data acquisition process. (**a**) Device wearing display, (**b**) BP measured by OMROM HEM-7211.

A total of 15 volunteers participated in the data collection of this experiment, including 13 males and 2 females, aged from 20 to 30 years old. These experiments were conducted according to the Helsinki declaration. All subjects volunteered to participate in the study and signed a written consent form before participation. Participants did not take any specific medications. During the acquisition, RA and LL electrodes were connected to the subject to collect II-lead ECG. In order to reduce the noise effect, an additional RL electrode was added as the reference ground. The PPG signal was collected at the index finger by the finger clip-type oximetry probe. Figure 2a illustrates an example of wearing the device during acquisition.

After the connection, volunteers were asked to sit in the chair with a relaxed posture and PPG and ECG signals were simultaneously collected for 30 s. Next, SBP and DBP values were obtained by measuring the left arm of the volunteers using a traditional cuff BP monitor (OMROM HEM-7211) after two minutes of rest, as shown in Figure 2b. This process was repeated five times with a 2 min interval between each measurement. The lowest and highest values of the five measurements were removed, and the mean value of the remaining three measurements was referred to as ground truth. Finally, a total of 90 records were saved as a private dataset.

### 2.3. MIMIC-III Dataset

MIMIC-III is an extensive and freely available database comprising health-related data associated with over forty thousand patients who stayed in critical care units of the Beth Israel Deaconess Medical Center between 2001 and 2012 (Johnson et al., 2016) [30]. It consists of a clinical database and a waveform database. The clinical database is a relational database composed of 26 tables and the clinical records can be queried by SQL language. The waveform database contains the waveform records, and the sampling rate was 125 Hz. Although the original database contained a large number of data records, many patients had incomplete information, which did not meet the experimental requirement, and so, we conducted a preliminary screening of the data. Furthermore, since the data in the collected database were from young people, we only retained the data of patients aged between 20 and 40 years to avoid bias in model training. Finally, we kept 900 records as a training data set.

### 2.4. Preprocessing

The same preprocessing procedure was performed for both the data in MIMIC-III and the collected data. ECG signal acquisition process was mixed with various interferences, such as baseline drift, electrode motion, muscle artifact, and power line interference [31]. We used a bidirectional infinite impulse response filter to denoise the ECG signal. Firstly, the mean value of the ECG signal was subtracted to remove the direct current component. Then, the fourth-order bidirectional low-pass filter was used to remove the high-frequency noise with a cut-off frequency of 35 Hz [32]. Finally, the second-order bidirectional high pass filter was used to remove the baseline drift with a cut-off frequency of 0.9 Hz.

To prevent waveform distortion caused by denoising, we used a low-pass filter and cubic spline interpolation to denoise the PPG signal. Firstly, the mean value of the PPG signal was subtracted to remove the direct current component, and then, the high-frequency noise was removed by using a low-pass filter with a cut-off frequency of 10 Hz. Finally, we used cubic spline interpolation to remove the baseline drift.

**Figure 3:**
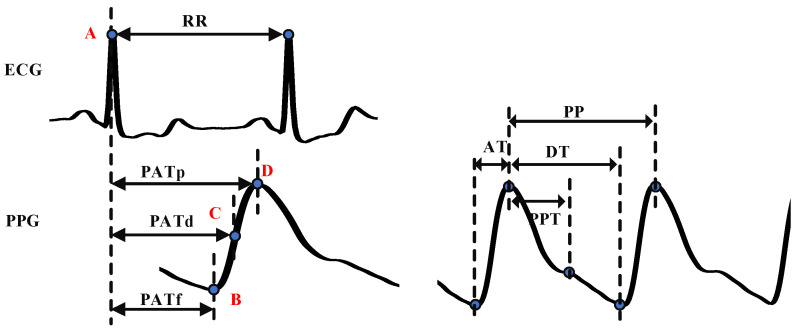
Schematic diagram of feature point detection.

MIMIC-III contains continuous ambulatory blood pressure waveforms, and so, we located the peaks and valleys of each segment as SBP and DBP labels, respectively. In order to facilitate subsequent feature extraction, we extracted corresponding feature points in ECG and PPG, respectively. Feature point detection mainly includes R-wave peak detection of the ECG signal, peak, valley, and maximum slope point detection of the PPG signal. In Figure 3, point A is the R-wave peak of the ECG signal. Points B, C, and D are the valley, maximum slope point, and the peak of the PPG signal, respectively. We used the Pan-Tompkin algorithm [33] to realize R-wave peak detection. Peak detection of the PPG signal was realized by the double-threshold detection algorithm [34]. Once the peak was determined, the search area of other feature points could be obtained. Starting from the peak, we searched forward the 0.4 times of the peak interval to find the zero-crossing point of the first derivative of the PPG signal from negative to positive, to realize the valley detection. The peak point of the first derivative was found in the area from the valley to the peak of the ascending branch of the PPG signal to detect the maximum slope point.

## 3. Proposed Method

**Figure 4:**
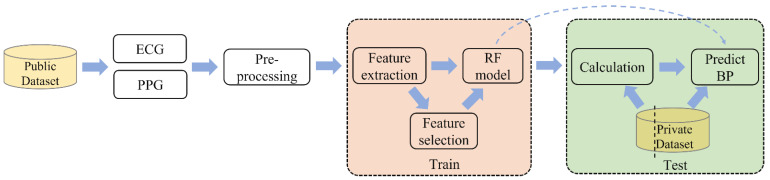
Block diagram of the proposed method.

In this section, we will introduce the process of feature extraction, selection, and BP prediction. Figure 4 demonstrates the block diagram of the proposed method.

### 3.1. Feature Extraction

We extracted 3 individual features and 22 waveform features. Table 1 lists the abbreviation and definition of features.

1. Individual features

It was proved that age and gender were correlated with arterial stiffness and PWV [35,36], and so, we took individual information into account. During the collection process, we recorded the gender, age, and weight information of each volunteer. Meanwhile, by querying the MIMIC-III clinical database, this information can also be obtained.

2. PAT

PAT is defined as the time required for blood to flow from the beginning of the electrical activation of the heart to the distal point [15]. As it contains the PTT feature, PAT is often used as a critical parameter for BP estimation [14]. Generally, the R- peak of the ECG signal is taken as the starting point, and the feature points of the PPG signal are taken as the ending point to calculate PAT. We extracted PAT_p_, PAT_f_, and PAT_d_, which means the distance from the R-peak of the ECG signal to the peak, the valley, and the maximum slope point of the PPG signal, which is shown in Figure 3.

3. Other time-related features

Heart rate (*HR*): *HR* reflects the heart cycle and can be calculated by the *RR* interval of the ECG signal. Cardiac output can be correlated with PTT through HR, and so, there is also a correlation between heart rate and blood pressure. HR can be calculated by the following formula:

(1) $$HR=\frac{60\times fs}{RR}$$

The peak-to-peak interval of the PPG signal (PP): PP is the time interval between two peaks. Studies found that participants with HPT or arteriosclerosis had “longer PP interval” than healthy participants [37].

The PPG waveform consists of ascending branches and descending branches. The ascending branch time (AT) is the time from the valley to the peak of the PPG signal, which was proven to be a useful feature for classifying the PWV. The descending branch time (DT) is the time from the peak to the next valley of the PPG signal.

Peak-to-peak time (PPT) is defined as the time between the first peak and the second peak or inflection point of the PPG signal. Its definition depends on the contour of the PPG waveform [38]. The second peak or inflection point is generated by reflection waves, which is related to the time required for the PPG signal to transit from the heart to the peripheral and return, so it can be used to evaluate the artery’s stiffness and the PWV.

4. Intensity-related features

From the perspective of the formation of BP, it is mainly affected by five factors: cardiac output, peripheral resistance, arterial wall elasticity, circulating blood volume, and blood volume ratio. PPG intensity ratio (PIR) is related to changes in arterial diameter, which is the main cause of peripheral resistance and blood volume. PIR can be used to evaluate the smooth muscle tension that regulates arterial blood pressure in the low-frequency range and improve the accuracy of blood pressure estimation [39,40]. We selected the ratio of peak intensity to valley intensity (PIRp) and the ratio of maximum slope point intensity to valley intensity (PIRmd), which can be calculated by the Formula (2).

(2) $$PIR_{x}=\frac{PI_{x}}{PI_{v}}$$

where *PIR_x_* is the PPG intensity ratio, *PI_x_* is the PPG intensity of the peak or the maximum slope point, and *PI_ν_* is the PPG intensity of the valley.

To reflect the change in the PPG intensity, we also added the statistical features of the PPG intensity, including the average value of the PPG intensity (PIavg), the standard deviation of the PPG intensity (PIsd), the maximum value of the PPG intensity (PImax), the minimum value of the PPG intensity (PImin).

5. *K* value

*K* value can reflect the physiological factors of the human cardiovascular system, such as vascular peripheral resistance, vascular wall elasticity, and blood viscosity. It is an important physiological index for the clinical examination of cardiovascular disease and has important clinical application value. It was calculated according to Formula (3):

(3) $$K=\frac{P_{m}-PI_{v}}{PI_{p}-PI_{v}}$$

where *P_m_* is the average intensity of the PPG signal in one cardiac cycle, which was calculated by Formula (4):

(4) $$P_{m}=\frac{1}{T}\int_{0}^{T}PI(t)dt$$

*PI*(*t*) is the intensity of the PPG signal at time *t*, and *T* is the time of one cycle of the PPG signal.

6. Other waveform features

The slope of ascending branch and descending branch can be calculated by *PI_p_*, AT, and DT. The area of ascending branch and descending branch can be obtained by integrating the PPG intensity. We added seven features to the model, including the ascending slope of the PPG signal (AS), the descending slope of the PPG signal (DS), the sum of ascending branch value of the PPG signal (SA), the average of ascending branch value of the PPG signal (AA), the sum of descending branch value of the PPG signal (SD), the average of descending branch value of the PPG signal (AD), and the sum ratio of the ascending branch value to the descending branch value of the PPG signal (SR).

### 3.2. Regression Model

Random forest (RF) [41] uses decision trees as weak learners and combines multiple decision trees to make predictions through random sampling with replacement. It can be used in classification and regression tasks, in which the final result is obtained by voting in classification and the average value is taken as the final result in regression. Even if there is a non-linear relationship between the input and label, it can still maintain good performance. Each decision tree constructed is different to reduce the deviation and variance of the prediction results. Because of the multiple combinations of the prediction results, RF is not sensitive to outliers and has a better ability for anti-overfitting and stability.

Compared with random search, grid search is time-consuming and easy to result in dimension disaster, and so, we used the bootstrap method to train the random forest. The number of decision trees was set to 100, the maximum depth of trees was set to 50, the min_samples_split was set to 2, and the min_samples_leaf was set to 1.

### 3.3. GCMI

MI is a measure of the mutual dependence between two random variables [42]. It measures the degree of information about one variable that is learned through observing the other [43]. It is a non-negative value, with higher values indicating stronger dependence between the variables. When two random variables follow the Gaussian distribution and X is a multidimensional vector, the calculated MI is the GCMI [29]. It can be obtained by the following formula:

(5) $$I\left(X,Y\right)=0.5\times \log_{2}[\frac{\left|\sum X\right|\left|\sum Y\right|}{\left|\sum XY\right|}]$$

where ∑X and ∑Y are the covariance matrices of *X* and *Y*, respectively, and ∑XY is the covariance matrix of the joint variables (*X*, *Y*). The detailed feature selection process is shown in Algorithm 1. First, each feature is calculated GCMI with SBP and DBP. If the value is zero, the corresponding feature will be removed. Due to the redundancy between features, the GCMI of the combined group may not necessarily be the highest. Therefore, we took the approach of deleting features and gradually eliminating the features that contributed the least to the group. Meanwhile, we set a threshold. When the minimum MI loss of the group exceeded the threshold after eliminating a feature, the feature selection process was stopped. The rest features were the filtered feature set.
**Algorithm 1.** GCMI-based feature selection method.$\mathrm{Input}:\;\mathrm{Training}\;\mathrm{set}\;D:F=\{f_{1},f_{2},\cdots ,f_{n}$}, the label COutput: Selected features F′Steps:(1) For i = 1 to n, do(2)    $\mathrm{If}\;I(f_{i}$
$,C)=0,\;\mathrm{then}\;F1_{\;}=F-f_{i}$
(3) end(4) Repeat (5)   Calculate I_1_ = I (F′, C)(6)   For i = 1 to n, do(7)        $F2_{\;}=F\prime -f_{i}$, Calculate I_2_ = I (F_2_, C)(8)   end(9)   $\mathrm{Find}\;\min\;(I_{2})\;\mathrm{with}\;f_{i}$, then(10)  $F\prime =F\prime -f_{i}$|min(I_2_)(11) Until I_1_ − min (I_2_) > 0.002(12) Return F′

## 4. Results

In this paper, the mean absolute error (*MAE*) and standard deviation (*STD*) are used to evaluate the prediction results, given by

(6) $$MAE=\frac{1}{N}\sum_{i}^{N}\left|{\tilde{y}}_{BP}^{i}-y_{BP}^{i}\right|$$

(7) $$STD=\sqrt{\frac{1}{N}\sum_{i=1}^{N}{\left({\tilde{y}}_{BP}^{i}-{\bar{y}}_{BP}^{}\right)}^{2}}$$

where *N* is the number of samples, *i* is one of the samples, ${\tilde{y}}_{BP}^{i}$, $y_{BP}^{i}$, and ${\bar{y}}_{BP}^{}$ are the reference *BP*, estimated *BP*, and average *BP*, respectively.

### 4.1. Waveform Display

**Figure 5:**
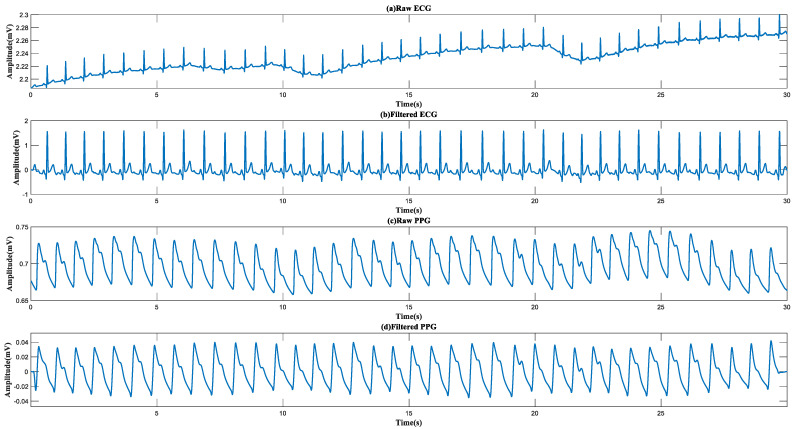
Raw and filtered PPG and ECG signals acquired from eMMC within 30 s.

Figure 5 shows the raw and filtered waveforms recorded within 30 s. It was observed that the raw ECG contained significant baseline drift due to respiration and body movement. After processing, the noise was removed, and the characteristic waveforms of the ECG, such as the P wave, QRS complex, and T wave, were well preserved. For PPG, with the use of the finger-clip blood oxygen probe, the shape of the original PPG signal was already very good, and the peak points, valley points, and dicrotic notch points were all very clear. High-quality signals provided a guarantee for the accurate extraction of more feature information. Moreover, comparing the ECG and PPG signals, they contained the same number of peaks during the same period, indicating that the recording of the signals was synchronous. It should be noted that due to PAT, there was a slight delay in their peak value of them. The waveform demonstrated that the entire hardware acquisition system was working well.

### 4.2. Feature Selection

**Figure 6:**
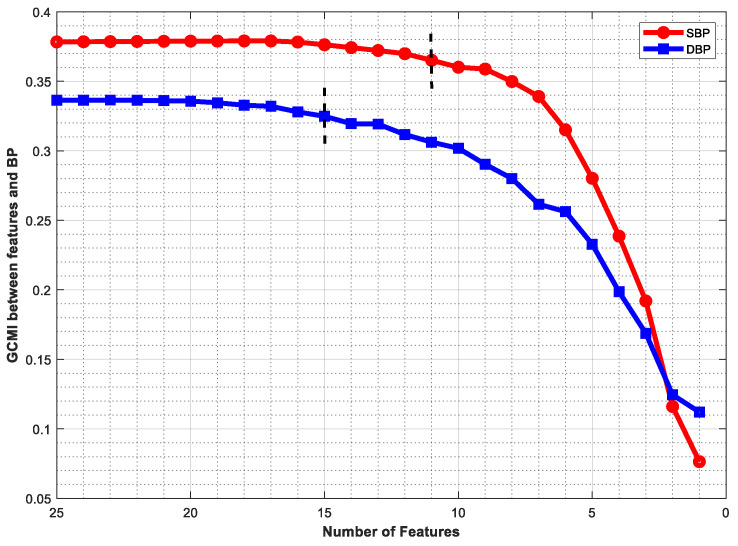
GCMI between different numbers of features and BP (the black dotted line represents the cut-off point for feature selection).

Figure 6 shows the GCMI between BP values and different feature sets. It can be seen that there was almost no change in GCMI when the features were initially deleted. This indicates that these features were redundant and the information they contained could be replaced by other features. As the number of features decreased, the GCMI started to decrease gradually. When the number of features of SBP and DBP was less than 11 or 15, respectively, the value of GCCI decreased significantly. Therefore, we chose these two values as thresholds for the feature subset. Unfortunately, if the number of features was less than 10, the GCMI values decreased rapidly for both SBP and DBP, suggesting that the feature set was no longer favorable for BP prediction at this time, because most information was lost.

### 4.3. Model Performance

According to the threshold, 11 and 15 optimal feature sets were retained for SBP and DBP, respectively, which are described in Table 2. It was found that almost all individual information was retained, which reflects that individual information plays an essential role in BP values. Meanwhile, from the definition, we can conclude that there was a certain similarity between the feature “PP” and “HR”. Only one of them was retained in the filtered feature set after screening.

The predicted results before and after feature selection are compared in Table 3. After feature selection, the MAE and STD were 7.93 ± 9.12 mmHg and 7.63 ± 8.61 mmHg, which was smaller than before. This indicates that there were abnormal features in the initial feature set, which negatively affected the robustness of the system. The selected feature set not only accelerated the training speed of the model but also improved its performance This also demonstrated the effectiveness of feature screening.

**Figure 7:**
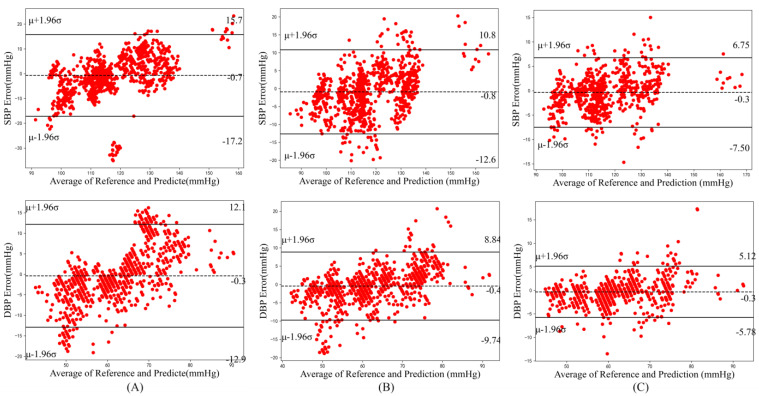
Bland–Altman plots showing the mean difference and 95% limits of agreement for SBP and DBP. The dotted line represents the mean value and the solid line represents the mean value ± 1.96 standard deviation. ((**A**) all features; (**B**) after feature selection; (**C**) after calculation).

Since the records in the training and testing sets were from different individuals, calibration was necessary for BP prediction. Based on the model trained on the training sets, we further selected a quarter of the testing sets to fine-tune the model, and used the calibrated model to predict the remaining data [44]. This operation is also known as personal calibration and is beneficial to reduce error. The result after calibration is shown in Table 4 and Figure 7. It can be seen that after calibration, the MAE and STD for SBP and DBP were significantly reduced, and both reached grade “A” on the criteria of British Hypertension Society Standard (BHS). This proves that after calibration, the optimal feature set achieved great performance.

To further analyze the results of this experiment, we displayed the Bland–Altman plots of the predicted SBP and DBP under different conditions. As shown in Figure 6, the feature selection proposed in this paper was effective by comparing A and B. The low and upper limits of agreement reduced from [−17.2, 15.7] mmHg to [−12.6, 10.8] mmHg for SBP and [−12.9, 12.1] mmHg to [−9.74, 8.84] mmHg for DBP. The calibration process helped it down further to [−7.5, 6.75] mmHg for SBP and [−5.78, 5.12] mmHg for DBP, respectively.

### 4.4. Comparison with Related Works

Many scholars conducted research on BP prediction based on feature extraction and selection from PPG and ECG signals. Table 5 provides details and the performance of these methods. Before calculation, although the prediction for DBP was slightly worse, the error for SBP in this paper was smaller than that of other methods. Furthermore, the calibration operation made up for this deficiency, and the results were further improved. After feature screening, the number of features retained were the least. However, in [45], the MAE for SBP and DBP was much smaller. On the one hand, the author trained and tested the model on a single dataset and the distribution difference of the data was small. On the other hand, the performance of Bi-GRU and attention mechanism was superior to traditional machine learning methods but also increased the computational complexity.

## 5. Discussion

Recently, wearable devices made great achievements in human multi-physiological signal detection [46,47]. The research of noninvasive continuous BP estimation based on the ECG signal and PPG signal attracted people’s attention, which makes the BP measurement equipment more portable and convenient. BP changes with time and is affected by multiple factors, such as exercise, age, emotion, diet, and temperature. It is easy to manifest the “white coat hypertension effect” when measuring BP in the hospital, which makes the accurate value more difficult to obtain [48]. Most databases such as UCI [15] contain multiple physiological signals, but information about age, gender, and weight is not available. Previous research had found that BP is highly related to these factors, which brings disadvantages to the research.

The designed device had the necessary functions for physiological signals acquisition, and the overall size was very small and convenient for daily signal acquisition. This provides a technical guarantee for our subsequent research without having to be limited by the public database. Meanwhile, as the number of extracted features grows, it is critical to obtain the most optimal set of features. Since the MI is nonlinear and simple to calculate [49], it can effectively represent the dependence between features and is suitable for BP prediction.

## 6. Conclusions

In this paper, we designed a wearable device to collect ECG and PPG signals. Then, we proposed a multi-parameter fusion method for noninvasive continuous BP estimation. GCMI method was introduced to reduce features from 25 to 11 and 15 for SBP and DBP, respectively. This procedure effectively avoided the influence of redundant features on the model. We trained the model on a public dataset and tested it on a private dataset. The results showed that after calibration, the proposed method achieved good results.

In the future, we intend collect more signals from the hospital or community, especially from the elderly over 60 years old with hypertension. The amount of current private datasets is still small and the source of data is concentrated in younger people. The old are at high risk for HPT and have a more complex physical situation. It is meaningful to improve the accuracy of continuous BP monitoring for the old. Furthermore, we attempted to combine the traditional features with the CNN and LSTM networks. The 25 features extracted in this paper were still limited. For information mining of big data, these networks have more advantages than manual extraction.

## Figures and Tables

**Table 1:** Feature abbreviation and definition.

Index	Feature	Definition
1	Gender	Gender of subjects
2	Age	Age of subjects
3	Weight	Weight of subjects
4	PATp	Pulse transit time from the R peak of ECG to the peak point of PPG
5	PATf	Pulse transit time from the R peak of ECG to the valley point of PPG
6	PATd	Pulse transit time from the R peak of ECG to the maximum difference point of PPG
7	HR	Heart rate
8	PP	The peak-to-peak interval of PPG
9	PPT	The time interval from the first peak point to the second peak or the inflection point of PPG
10	PIRp	PPG intensity ratio of the peak point to valley point
11	PIRmd	PPG intensity ratio of the maximum difference point to the valley point
12	PIavg	The average amplitude of PPG
13	PIsd	The standard deviation of PPG
14	PImax	The maximum amplitude of PPG
15	PImin	The minimum amplitude of PPG
16	SA	The sum of ascending branch value of PPG
17	AA	The average of ascending branch value of PPG
18	SD	The sum of descending branch value of PPG
19	AD	The average descending branch value of PPG
20	SR	The sum ratio of ascending branch value to descending branch value of PPG
21	K	K value of PPG
22	AT	Ascending time of PPG
23	DT	Descending time of PPG
24	AS	The ascending slope of PPG
25	DS	The descending slope of PPG

**Table 2:** Optimal feature subset for SBP and DBP.

	SBP			DBP		
	Age	AA	DS	Gender	HR	AD
	Weight	SD		Age	K	SR
Feature	PATf	AD		Weight	PIsd	AT
	HR	SR		PATf	AA	AS
	PIsd	AT		PATd	SD	DS

**Table 3:** Results before and after feature selection.

	Feature Selection	MAE ± STD (mmHg)
SBP	No	9.12 ± 9.83
	Yes	7.93 ± 9.12
DBP	No	8.31 ± 9.23
	Yes	7.63 ± 8.61

**Table 4:** The MAE and STD for SBP and DBP after calibration.

	MAE ± STD	The Proportion of MAE			Grade
	(mmHg)	≤5 mmHg	≤10 mmHg	≤15 mmHg	
SBP	5.21 ± 5.98	65%	86%	97%	A
DBP	4.15 ± 5.66	72%	89%	98%	A
	-	60%	85%	95%	A
BHS	-	50%	75%	90%	B
	-	40%	65%	85%	C

**Table 5:** Performance comparison with related works.

Calculation	Works	Feature	Methods	SBP (mmHg)		DBP (mmHg)	
		Num.*		MAE	STD	MAE	STD
No	Ours	11/15 *	RF	7.93	9.12	7.63	8.61
	Kachuee [15]	15/15	Adaboost	11.17	10.09	5.35	6.14
	Zhang [19]	20/20	Adaboost	10.03	14.43	5.35	8.33
Yes	Ours	11/15	RF	5.21	5.98	4.15	5.66
	Zhang [19]	20/20	Adaboost	7.73	7.96	4.30	4.50
	Geerthy [20]	23/17	RF	9.00	-	5.48	-
	Kachuee [15]	15/15	Adaboost	8.21	5.45	4.31	3.52
	El-Hajj [45]	22/22	Bi-GRU	2.58	3.35	1.26	1.63

*: The number before and after “/” is the feature number for SBP and DBP respectively.

## Data Availability

The data presented in this study are available from the corresponding author upon reasonable request.
